# Multilingual assessment of early child development: Analyses from repeated observations of children in Kenya

**DOI:** 10.1111/desc.12875

**Published:** 2019-07-28

**Authors:** Heather A. Knauer, Patricia Kariger, Pamela Jakiela, Owen Ozier, Lia C.H. Fernald

**Affiliations:** ^1^ Berkeley School of Public Health University of California Berkeley California; ^2^ Center for Effective Global Action University of California Berkeley California; ^3^ Center for Global Development Washington District of Columbia; ^4^ Human Development Team The World Bank Development Research Group Washington District of Columbia; ^5^ Present address: School of Social Work University of Michigan Ann Arbor Michigan

**Keywords:** BPVS, language of instruction, MDAT, multilingual environments, PPVT, school readiness

## Abstract

In many low‐ and middle‐income countries, young children learn a mother tongue or indigenous language at home before entering the formal education system where they will need to understand and speak a country's official language(s). Thus, assessments of children before school age, conducted in a nation's official language, may not fully reflect a child's development, underscoring the importance of test translation and adaptation. To examine differences in vocabulary development by language of assessment, we adapted and validated instruments to measure developmental outcomes, including expressive and receptive vocabulary. We assessed 505 2‐to‐6‐year‐old children in rural communities in Western Kenya with comparable vocabulary tests in three languages: Luo (the local language or mother tongue), Swahili, and English (official languages) at two time points, 5–6 weeks apart, between September 2015 and October 2016. Younger children responded to the expressive vocabulary measure exclusively in Luo (44%–59% of 2‐to‐4‐year‐olds) much more frequently than did older children (20%–21% of 5‐to‐6‐year‐olds). Baseline receptive vocabulary scores in Luo (β = 0.26, SE = 0.05, *p* < 0.001) and Swahili (β = 0.10, SE = 0.05, *p* = 0.032) were strongly associated with receptive vocabulary in English at follow‐up, even after controlling for English vocabulary at baseline. Parental Luo literacy at baseline (β = 0.11, SE = 0.05, *p* = 0.045) was associated with child English vocabulary at follow‐up, while parental English literacy at baseline was not. Our findings suggest that multilingual testing is essential to understanding the developmental environment and cognitive growth of multilingual children.

**Research Highlights**

- This study measured vocabulary among Kenyan children 2–6 years old, at two time points, across three languages: Luo (mother tongue), Swahili and English (official languages).
- During testing, the youngest children strongly preferred to express themselves in Luo, whereas older children were more likely to respond in Luo and English.
- Luo receptive vocabulary among all children at baseline was significantly associated with English receptive vocabulary at follow‐up, even accounting for baseline English and Swahili.
- Baseline caregiver literacy in Luo, rather than English, was robustly related to children's later receptive vocabulary in English.

## 1 INTRODUCTION

Up to 40% of children worldwide speak a mother tongue that is not used at school (Clegg & Simpson, 2016; Walter & Benson, 2012), which results in significant, negative consequences for hundreds of millions of children (Ball, 2011). For example, in a global study of fourth‐grade students, children whose home language differed from the testing language were 10% less likely to achieve the most basic level of reading proficiency compared to students who spoke the testing language at home (UNESCO, 2018). Though mother tongue instruction is potentially complicated to implement in linguistically diverse environments, it may allow children to learn more and may better permit their parents to engage with teaching materials and monitor student performance (Benson, 2002; Konsonen, 2005; Lieberman, Posner, & Tsai, 2014). In the case of Kenya, 45% of mothers of school‐aged children cannot read English at a second‐grade reading level (Uwezo, 2015); in one study, 72% of parents reported not understanding how to interpret student‐learning data (Lieberman et al., 2014). Thus, a country's policy regarding language of instruction (LOI) can have significant implications for children's development in ways that interact with poverty, parental literacy, ethnicity, and other risk factors faced by vulnerable children as they move through the formal education system.

### 1.1 Child assessment in multilingual environments

Child development assessments allow teachers to understand how and what children are learning, to diagnose learning differences or language disorders, and to benchmark achievement against national or international standards (Armon‐Lotem, de Jong, & Meir, 2015; Snilsveit et al., 2016). Similarly, researchers and policymakers rely on child assessments to examine programme effectiveness. In both academic and nonacademic settings, students are routinely tested in only one language, either the LOI or parents’ preferred language. It is challenging to assess child development, language disorders, and school readiness in such populations, both because these children develop linguistic skills in multiple languages simultaneously and because most widely used measures of child development have not been validated in local languages and low‐ and middle‐income country (LMIC) contexts. Many assessments created and validated in U.S. or European samples do not demonstrate the same strong psychometric characteristics when applied in different settings (Fernald, Prado, Kariger, & Raikes, 2017). To capture the linguistic development of children in LMIC contexts, it is crucial to adapt, or develop, and subsequently validate assessments in children's mother tongues (Prado et al., 2018).

Child development assessments conducted in a single language may not fully reflect a multilingual child's developmental outcomes and learning trajectory (Cummins, 1979, 2001; Peña, Bedore, & Kester, 2015). Bilingual children's conceptual vocabularies are similar in size to those of monolingual children; however, their vocabulary size in each language is smaller than that for monolingual children (Bialystok, Luk, Peets, & Yang, 2010; Hammer et al., 2014). The amount of overlap in children's vocabulary between the two languages may depend on how typologically related the two languages are (Hammer et al., 2014). Furthermore, bilingual children's performance on language assessments in their second language may have more to do with exposure to the second language than knowledge transfer based on first‐language proficiency (Keller, Troesch, & Grob, 2015). For this reason, children may perform better on certain aspects of the tests, such as letter sounds, syllables, or reading fluency, when they are tested in the LOI as compared to their native language (Bialystok, Majumder, & Martin, 2003). Greater reading fluency or decoding skills in the LOI, however, do not necessarily indicate that children have greater reading comprehension in the LOI (Piper, Schroeder, & Trudell, 2016; Piper, Zuilkowski, & Ong'ele, 2016). For multilingual children, assessment of language and other domains of development should account for all of the child's languages (Pearson, Fernandez, & Oller, 1993). Furthermore, the assessment should ideally capture the complexity of the child's language environment or the extent to which a child's language is specific to a certain context (i.e. school, home, or community) (Pearson et al., 1993; Toppelberg & Collins, 2010). To date, most studies of bilingual or multilingual child language development have been conducted in high‐income countries (Barac, Bialystok, Castro, & Sanchez, 2014), although a few studies have been conducted in sub‐Saharan Africa (e.g. Alcock, 2017; Alcock & Alibhai, 2013; Alcock, Holding, Mung'ala‐Odera, & Newton, 2008; Cockcroft, 2016; Demuth, 2003; Potgieter & Southwood, 2016). Thus, limited data are available to help us understand young children's verbal development in LMIC contexts.

### 1.2 Current approaches to child assessment across contexts

**Figure 1:**
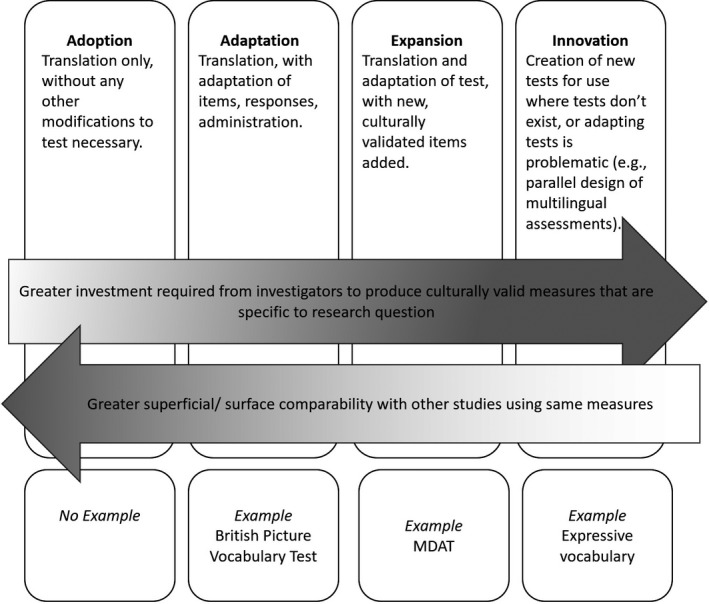
Proposed strategies for measuring early child development in Western Kenya, with examples from study

There is an inherent tension between the desire to employ widely used, well‐validated measures and the need to adapt items to local contexts. Assessments that are well validated in one context but not appropriately adapted for another may not maintain their properties (Peña, 2007) and may perform unreliably (Gibson, Jamulowicz, & Oller, 2017). This problem is particularly pronounced for tests designed and validated in high‐income countries that, without thorough and careful adaptation, often generate items poorly suited to a LMIC context (Fernald et al., 2017; van de Vijver & Poortinga, 2005). Investigators generally have four approaches when using a measure in a new country or context: adoption (translation of an existing test without modification); adaptation (translation with careful modification of items, responses, and administration); expansion (adding items to an existing test to suit a particular cultural or linguistic context); or creation of new tests **(**Figure 1). These approaches have been used in the LMIC context (He & van de Vijver, 2012; Weber, Fernald, Galasso, & Ratsifandrihamanana, 2015) and in higher income contexts, where the parallel design of assessments is necessary to simultaneously test children's verbal development across multiple languages (Haman, Łuniewska, & Pomiechowska, 2015). When multiple tests are needed to comprehensively measure various capacities, a more diversified strategy may be to adopt some tests, adapt others, expand an existing test to include new test items, and create new tests that are internally valid for the context.

### 1.3 Language policy in the African context

**Figure 2:**
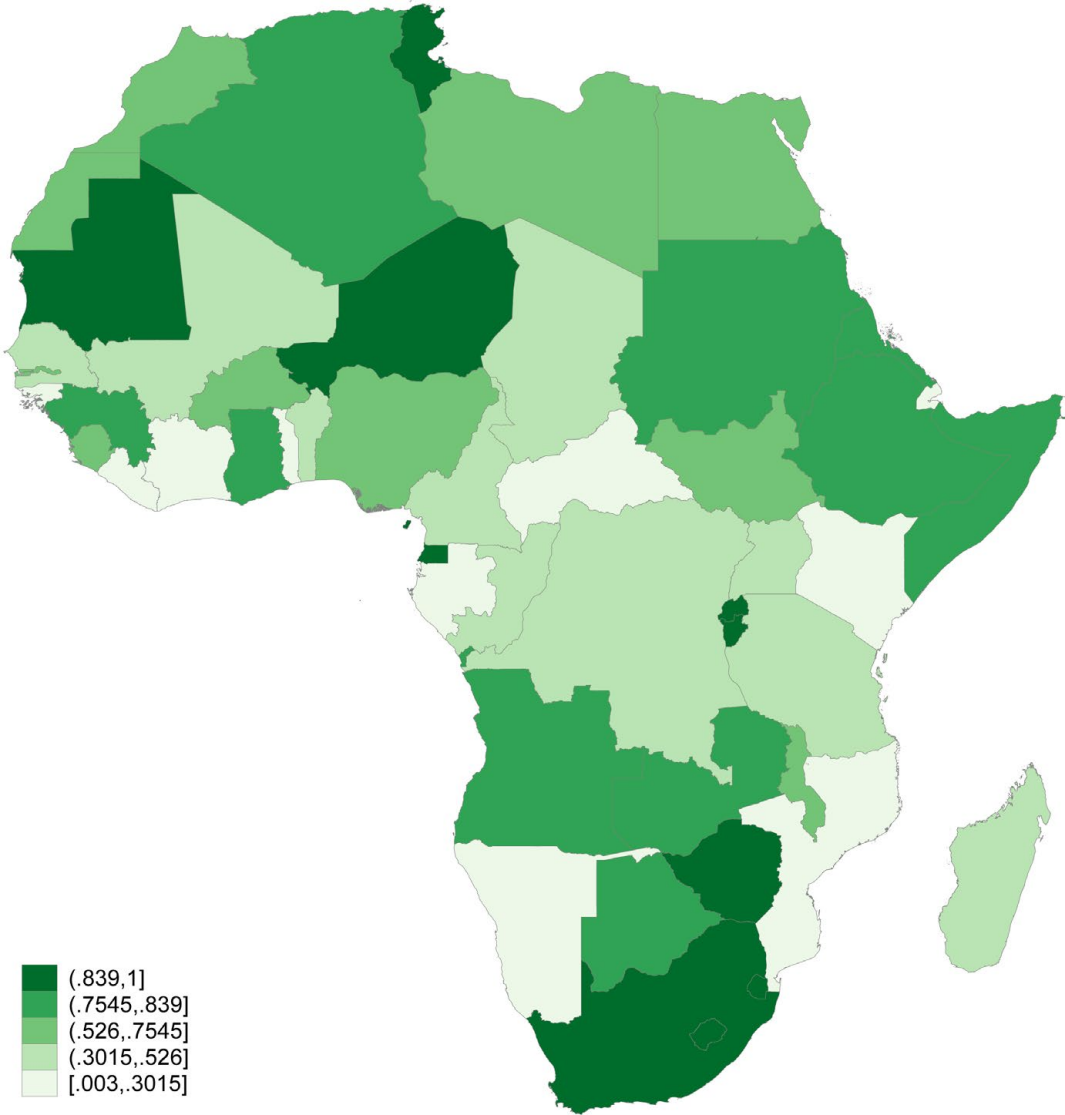
Proportion of population speaking a native language used in any official capacity. Notes. Figure shows proportion of population whose native language is classified as an “institutional” language in the ethnologue (Lewis et al., 2016). Institutional languages include national and provincial languages (used in government), languages other than national and provincial languages that are used in institutional education, and languages used for “wider communication” through mass media

Over 2,149 mother tongue languages are spoken in Africa (Lewis, Simons, & Fenning, 2016), and more than a quarter of the African population speaks a native language that is not in official use in the educational system or by the government (Figure 2; Lewis et al., 2016). In spite of UNESCO's recent call for at least 6 years of mother tongue education (UNESCO, 2017), there are several reasons for resistance to mother tongue instruction. For example, parents and teachers sometimes believe that children who learn in the mother tongue language will fall behind those who learn in English (Jones, 2012; Trudell, 2007). In addition, linguistically appropriate teaching materials are not always available (Musau, 2003; Waithaka, 2017), and teachers may not be fluent in the local mother tongue (Manyonyi, Mbori, & Okwako, 2016; Trudell & Piper, 2014). The misalignment between children's first languages and those used in schools has important implications for the assessment of school readiness and learning outcomes: namely, children from linguistically marginalized families risk being underserved by the educational system.

### 1.4 Current study

Our study took place in the Luo‐speaking region of western Kenya, a country with 68 spoken languages (Lewis et al., 2016). English and Swahili are the official languages (i.e. for all government proceedings and publications), but literacy rates in these languages, while perhaps relatively high within sub‐Saharan Africa, are still quite low. For example, only 55% of mothers of school‐aged children, and about 51% of children aged 7–13 years can read English at a second‐grade level (Uwezo, 2015). In our study area, only 31% of young primary school students are taught in Luo, while the rest are taught in either English or Swahili (Piper & Miksic, 2011).

The purpose of this study was to compile a set of child development assessments to evaluate the effects of a literacy promotion programme on multilingual children's development. Our first aim was to validate language assessments for children aged 2–6 years. Our second aim was to understand children's performance on receptive vocabulary assessments in mother tongue (Luo) and official languages (English and Swahili), and the extent to which scores on each of these assessments were associated with children's receptive and expressive vocabulary at a 5‐ to 6‐week follow‐up. We hypothesized that baseline scores in all languages, but especially English, would be significantly associated with child English receptive vocabulary at follow‐up, as they all measure aspects of language skill. Our final aim was to examine the relationship between caregiver literacy––in both the mother tongue (Luo) and the LOI (English)––and child receptive and expressive vocabulary and to test whether the strength of the association between mother tongue and LOI literacy varied with caregiver literacy. We focused primarily on children's English vocabulary at follow‐up as an indicator for school readiness, as this is the LOI at higher grade levels and the de facto language of instruction for many young children in our study area. We hypothesized that caregiver literacy in both languages at baseline would be significantly associated with child receptive and expressive language at follow‐up, and that the association between the baseline measure of child receptive vocabulary in Luo and English vocabulary at follow‐up would be strongest among children whose parents had lower English literacy.

## 2 METHODS

### 2.1 Study design and sample description

The measures described in this paper were developed for an ongoing cluster‐randomized trial in Kenya's Kisumu and Homa Bay Counties that is designed to evaluate the effectiveness of a book distribution and parenting training programme on child development (see trial registry: https://doi.org/10.1186/ISRCTN68855267 and pilot results: Knauer, Jakiela, Ozier, Aboud, & Fernald, in press). Families with at least one child between the ages of 24 and 83 months were recruited from a set of nine primary school catchment areas in rural communities within two hours’ drive from Kisumu. A total of 357 primary caregivers (one per household) and 510 children were assessed during household visits (average 1.43 children per household); five child assessments were incomplete, resulting in an analysis sample size of 505. A total of 442 children were assessed at follow‐up (5–6 weeks later), with 68 children lost due to relocation or difficulty in making contact.

### 2.2 Measures

#### 2.2.1 Overview of child assessments

To develop our test battery, we used adoption, adaptation, expansion, and creation of new tests for different developmental domains. All assessments were translated to Swahili or Luo and then back‐translated to English by a different team of translators (two for each language) who did not have access to the original measure. The first and second authors (HK and PK) then met with a group of translators and discussed each translation to ensure that words conveying the desired meaning were chosen over direct translation (In Swahili and Luo, several words were often possible depending on the intent of the item). The assessments were then pretested, and any additional study team concerns or discrepancies were addressed. Items for the vocabulary assessments were ordered by difficulty, as measured in a small pilot sample (between 30 and 61 respondents).

The assessors hired to administer the tests in the current study had university degrees, were from the study area, spoke Luo as their mother tongue, and were trained on the full battery of tests by the first and second study authors. On a subset of 48 children, two assessors double coded the baseline assessment to assess interrater reliability (IRR) for each of the assessments (Table S1).

#### 2.2.2 Receptive vocabulary

We created receptive vocabulary assessments based on the British Picture Vocabulary Scale III (BPVS III) (Dunn, Dunn, & Styles, 2009), which includes 168 items for individuals 3–16 years old (see details of translation and adaptation in Appendix A). Knowledge of words is measured by asking the respondent to point to one of four pictures that corresponds to a word (object, person, or action) spoken by the assessor. The BPVS has been adapted for use in South Africa (Cockcroft, 2016) and Indonesia (Prado, Alcock, Muadz, Ullman, & Shankar, 2012) and is the British adaptation of the Peabody Picture Vocabulary Test (Dunn & Dunn, 1997), which has also been used in neighboring areas of Kenya (Ozier, 2018). As we wanted to capture young children's knowledge of Luo, Swahili, and English words, we created three sets of nonoverlapping words of varying difficulty, with 27 Luo items, 32 Swahili items, and 34 English items. Administration ended when a child failed six out of a set of eight items.

#### 2.2.3 Expressive vocabulary

We developed our own measure of expressive vocabulary after reviewing various expressive vocabulary tests and concluding that the stimulus words and/or pictures were not appropriate to the context (see details in Appendix B **)**. The assessment was a picture‐naming task, in which children were presented with flash cards bearing a single illustrated stimulus item or object (noun) per card and were asked in the child's preferred language, “What is this?” for each item. Children were not instructed as to which language to respond in, but responses in any language were accepted. We did not provide further instruction because code‐switching during conversation is common in this area, and very young children may not be aware which language they are actually speaking for a given word. Thus, a child could respond to each item in the 20‐item test in English, Luo, or Swahili to score a pass for expressing the word verbally. Administration ended with three consecutive fails.

#### 2.2.4 Other child‐level assessments

The Malawi Developmental Assessment Tool (MDAT) was created and validated for use in rural Malawi with children 1–84 months of age (Gladstone et al., 2010). It includes four 34‐item subscales (fine motor/perception, language/hearing, gross motor, social‐personal), with many items adapted from existing Western tests (see details of our adaptation in Appendix C). The MDAT is currently being used in various countries, including Mali, Sierra Leone, Rwanda, Burkina Faso, and Zimbabwe (M. Gladstone, Pers Commun., June 24, 2016). The western Kenya adaptation was initiated by the first, second, and fifth authors (HK, PK, and LCHF) for the Kenya Life Panel Survey (e.g. Baird, Hicks, Kremer, & Miguel, 2016), a longitudinal study that examines the intergenerational effects of health investments. We used the translations and piloting data from that study to further adapt and expand the language and fine motor/perception subtests of the MDAT for this study. The final adapted language test had 26 items. To further reduce the overall length of the test, we created start and stop rules for three different age groups (24–35 months; 36–59 months; 60–71 months) based on pass rates during piloting.

#### 2.2.5 Caregiver survey

Data were gathered on household assets, housing quality, household size and composition, and the age and education level of primary caregivers. In addition, we assessed caregiver literacy by asking caregivers to read a simple, five‐word (second‐grade level) sentence in each language adapted from the Early Grade Reading Assessment (EGRA; Gove & Wetterberg, 2011). Caregivers who read more than one word incorrectly in all three languages were categorized as illiterate. Working memory in caregivers was assessed using a summary score of the forward and backward digit span test (Ozier, 2018; out of 20 possible), and mental health was measured using an adapted version of the Centers for Epidemiological Studies‐Depression scale (CES‐D; Radloff, 1977; scores range 0–60). Household support for learning was measured with a set of items drawn from the HOME Inventory, Family Care Indicators, and UNICEF MICS4 (Bradley, Corwyn, McAdoo, & Coll, 2001; Hamadani et al., 2010; Kariger et al., 2012).

### 2.3 Statistical analysis

To address the first aim of validating our assessments by examining their psychometric properties, we measured: (a) the internal consistency of the measures using Cronbach's alpha; (b) IRR using Cohen's kappa, Krippendorff's alpha, and percent agreement; (c) construct validity by examining the correlations between the measures; and (d) convergent validity by examining associations with known covariates in bivariate regressions. For our second aim, to better understand the relationships between baseline measures of mother tongue and LOI receptive vocabulary and scores on subsequent vocabulary assessments, we estimated a series of ordinary least squares (OLS) regression models to examine the associations between baseline age‐standardized receptive vocabulary scores in all languages (English, Swahili, and Luo) and English receptive vocabulary at follow‐up. We repeated this analysis for follow‐up measures of child expressive vocabulary as well as Swahili and Luo receptive vocabulary; we present these results as supplemental analyses. Our final aim was to examine the association between caregiver literacy and child vocabulary at two time points. We used OLS regression to examine the association between baseline caregiver literacy in Luo and English and child English and Luo receptive and expressive vocabulary scores at follow‐up. To test whether the relationship between baseline mother tongue and LOI receptive vocabulary scores and follow‐up LOI receptive vocabulary varied with caregiver literacy, we estimated OLS regression models that included both caregiver literacy and baseline child receptive vocabulary (in English, Luo, and Swahili). All regressions used age‐adjusted *z‐*scores for child vocabulary, and standard errors were adjusted for household clustering. All statistical analyses were conducted using Stata 14.2.

## 3 RESULTS

### 3.1 Descriptive statistics

The average age of children in the study was 54.42 months (range 24–83 months) (Table 1). About one‐quarter (27%) of caregivers were illiterate. Maternal and household characteristics were similar to those observed in the representative 2014 Kenya Demographic and Health Survey sample for the study area.

**Table 1:** Descriptive characteristics of children, caregivers, and households at baseline

	Obs.	Mean	*SD*	Min.	Max.
*Child characteristics:*					
Child age in months	505	54.42	17.51	24	83
Child is male	505	0.53	0.50	0	1
Height‐for‐age *z‐*score	489	−0.23	1.40	−4.58	4.39
Child is stunted (HAZ ≤2SD)	489	0.08	0.27	0	1
*Primary caregiver characteristics:*					
Caregiver is child's mother	353	0.85	0.36	0	1
Caregiver is child's father	353	0.02	0.14	0	1
Caregiver is child's grandmother	353	0.11	0.31	0	1
Caregiver mother tongue is Luo	353	0.95	0.23	0	1
Caregiver education: no formal schooling	353	0.02	0.15	0	1
Caregiver education: incomplete primary school	353	0.48	0.50	0	1
Caregiver education: completed primary, not secondary	353	0.41	0.49	0	1
Caregiver education: completed secondary school	353	0.08	0.28	0	1
Caregiver illiterate	353	0.27	0.45	0	1
Caregiver working memory (out of 20)	353	2.43	1.74	0	9
Caregiver depressive symptoms (out of 26)	346	10.33	5.36	0	26
*Household characteristics:*					
Household size	353	3.03	0.85	2	6
Any children's books in the home	353	0.13	0.34	0	1
Number of children's books in the home	353	0.22	0.71	0	5
Someone has read to the child in past 3 days	353	0.48	0.50	0	1
Family care indicators score (out of 17)	353	7.95	3.58	0	17
Rooms per person	353	0.89	0.43	0.20	3.00
Household has cement floor	353	0.24	0.43	0	1
Household has iron roof	353	0.96	0.20	0	1
Household has electricity	353	0.30	0.46	0	1
Household has latrine	353	0.69	0.46	0	1
Household wealth index	353	0.04	2.22	−3.51	9.80

Notes

Summary statistics on 505 children and 353 caregivers for whom baseline data are available. Baseline height data is missing for 16 children. Seven caregivers declined to answer the questions on depressive symptoms.

### 3.2 Psychometric properties of the instruments

The internal consistency of the vocabulary measures ranged from *α* = 0.57–0.90: Cronbach's alphas were lowest for the expressive and English receptive tests and highest for the MDAT language test (Table S1). The internal consistency of the receptive vocabulary assessments was higher for Luo (*α* = 0.78) and Swahili (*α* = 0.76) than for English (*α* = 0.57). The IRR of the receptive vocabulary tests was *κ* = 1 for Luo,* κ* = 0.89 for Swahili, and* κ* = 0.95 for English. The internal consistency of the expressive vocabulary test was *α* = 0.67, while the IRR was *κ* = 0.95. The internal consistency of the MDAT fine motor and language tests was *α* = 0.94 and *α* = 0.90, respectively. IRR of the total score for each measure was *κ* = 0.93 for fine motor and *κ* = 0.86 for language.

Correlations among the baseline child development assessments ranged from *r* = 0.32–0.56, and all correlations were statistically significant at the *p* < 0.001 level (Table S2). The three age‐normalized receptive vocabulary scores were all moderately correlated with each other, the expressive vocabulary score, and the MDAT scores, while the expressive vocabulary score was also moderately correlated with both MDAT scores. The MDAT tests had the strongest correlation (*r* = 0.56) with each other; among the vocabulary assessments, they had the strongest correlations with Luo vocabulary (*r* = 0.48–0.49).

The associations between baseline child, caregiver, and household characteristics with child age‐adjusted child development scores are presented in Table S3. In bivariate regression analyses adjusted for household clustering, child height‐for‐age *z‐*score was significantly associated with all child development assessments (*β* = 0.25–0.33, *SD* = 0.03–0.04, *p* < 0.001 for all). Caregiver characteristics (education, literacy, and cognition) were most strongly associated with child expressive vocabulary and MDAT scores, while caregiver depressive symptoms were not associated with any child assessments. Finally, household characteristics were not consistently associated with child assessments.

### 3.3 The role of language in child development assessment

**Figure 3:**
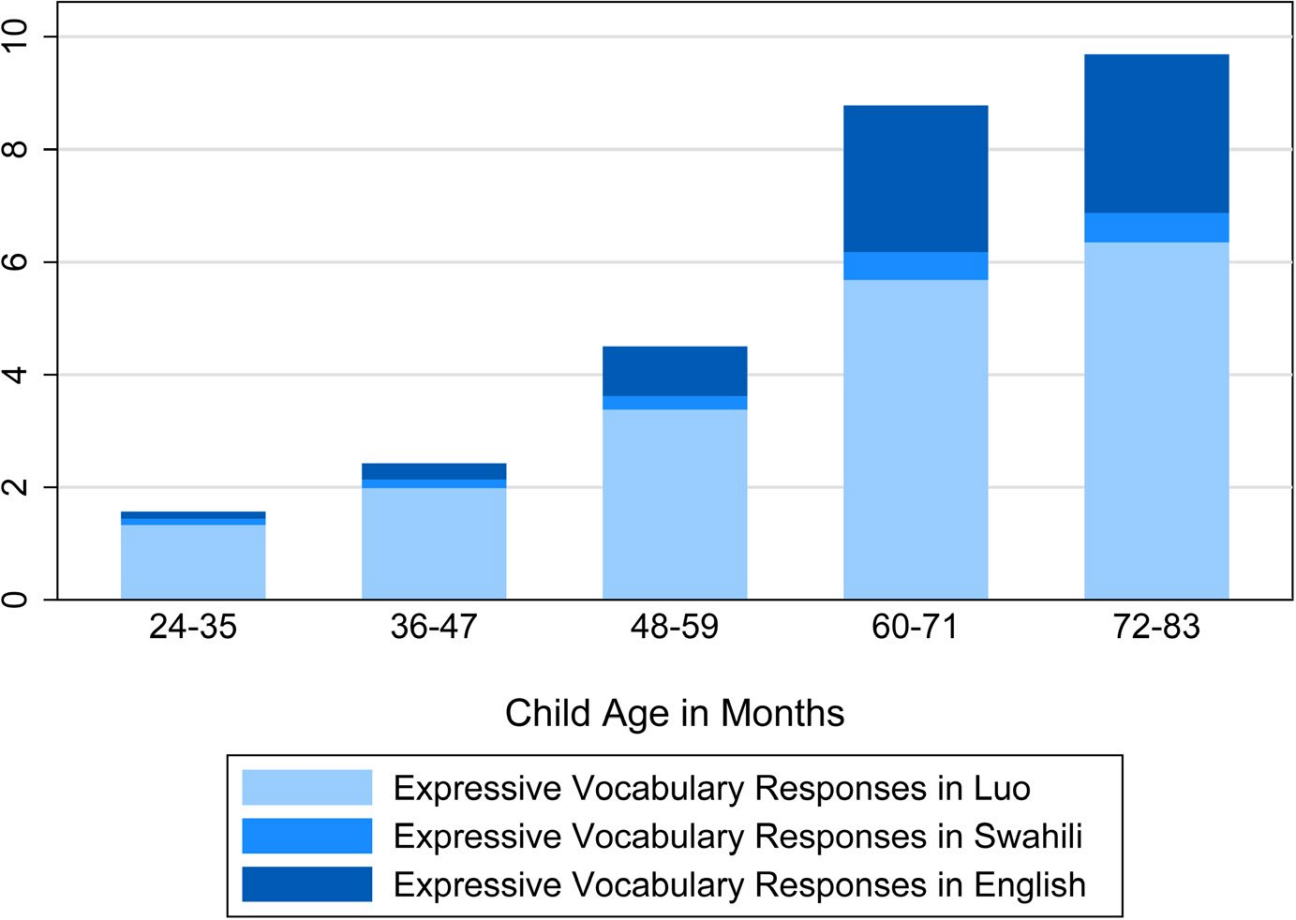
Number of expressive vocabulary responses in each language, by child age

**Figure 4:**
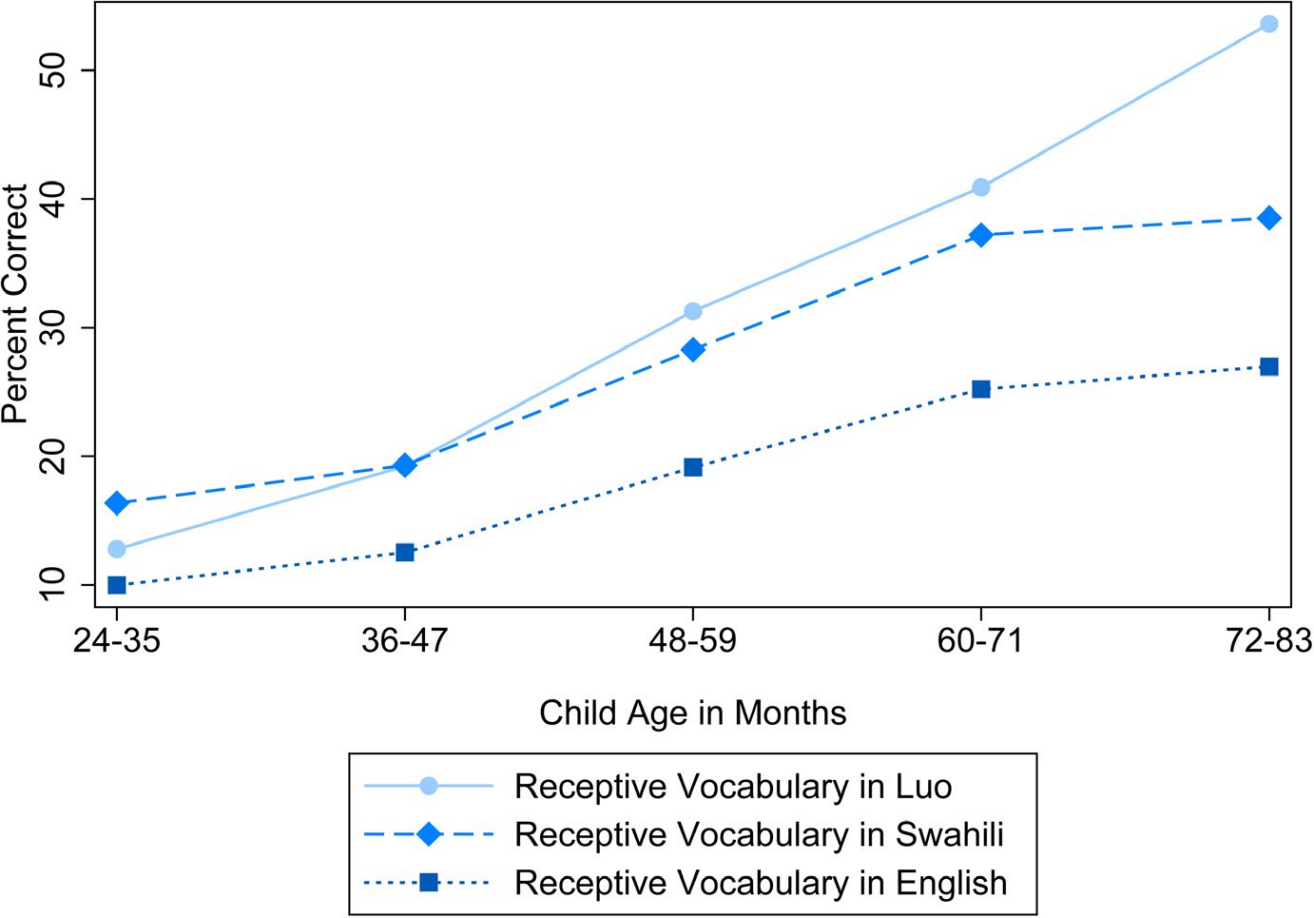
Receptive vocabulary test performance, by language, and child age

At baseline, 2‐year‐old children knew, on average, 3.45 of 27 (*SD* = 3.40) Luo receptive vocabulary words, 3.40 of 34 (*SD* = 3.41) English receptive vocabulary words, 5.08 of 31 (*SD* = 4.20) Swahili receptive vocabulary words, and 1.71 of 20 expressive vocabulary words in any language (*SD* = 2.02) (Table 2). Children's vocabulary progressed with age (Figures 3 and 4), such that 6‐year‐olds knew, on average, 14.48 (*SD* = 5.39) Luo, 9.17 (*SD* = 3.70) English, and 11.94 (*SD* = 5.43) Swahili receptive words, and 9.78 expressive words in any language (*SD* = 5.83).

**Table 2:** Descriptive statistics of child measures at baseline

	2 years		3 years		4 years		5 years		6 years		Overall
	*N* = 106		*N* = 76		*N* = 110		*N* = 113		*N* = 105		*N* = 505
	mean	sd/n	mean	sd/n	mean	sd/n	mean	sd/n	mean	sd/n	%
Receptive vocabulary											
English (out of 34)	3.40	3.41	4.26	3.44	6.51	3.76	8.58	3.92	9.17	3.70	
Swahili (out of 31)	3.45	3.40	5.19	3.96	8.44	4.81	11.04	5.00	14.48	5.39	
Luo (out of 27)	5.08	4.20	5.99	3.87	8.77	3.87	11.53	4.90	11.94	5.43	
Expressive vocabulary (out of 30)	1.71	2.02	2.60	2.37	4.82	3.86	8.73	5.33	9.78	5.83	
% of responses in English	4.44%	15.29%	6.42%	16.30%	15.57%	24.08%	28.34%	26.04%	29.02%	27.10%	19.21%
% of responses in Swahili	7.02%	25.54%	4.90%	18.24%	5.03%	16.13%	4.83%	12.97%	3.80%	7.28%	4.96%
% of responses in Luo	88.54%	28.78%	88.68%	24.97%	79.39%	30.25%	66.83%	29.00%	67.18%	29.13%	75.83%
% answered only in English	0.94%	1	0.00%	0	2.73%	3	3.54%	4	4.76%	5	2.55%
% answered only in Swahili	3.77%	4	1.32%	1	0.91%	1	0.00%	0	0.00%	0	1.18%
% answered only in Luo	44.34%	47	59.21%	45	47.27%	52	21.24%	24	20.00%	21	37.06%
Multiple language response	50.94%	54	39.47%	30	49.09%	54	75.22%	85	75.24%	79	59.22%
Adapted MDAT fine motor (out of 43)	5.52	3.62	12.99	5.34	18.34	4.85	22.65	4.85	25.98	5.05	
Adapted MDAT language (out of 36)	10.22	1.93	13.81	3.28	16.93	3.27	20.78	2.92	22.22	2.64	

Notes

English, Swahili, and Luo vocabulary are raw total receptive vocabulary scores, measured using three assessments based on the British Picture Vocabulary Scale (BPVS). Expressive vocabulary raw scores were measured using a tool developed from the PPVT. Percent (%) English, Swahili, and Luo expressive are the mean (and sd) percent of responses given in each language for the expressive vocabulary test. Only in English, Swahili, and Luo are the percent (and n) of children who answered the expressive vocabulary exclusively in each language. Multiple language response are the percent (and n) of children who answered in more than one language. The adapted MDAT are the raw scores from the Kenya adaptation of the Malawi Developmental Assessment Tool (MDAT).

Overall, 189 children answered the expressive vocabulary test entirely in Luo, while 13 children answered entirely in English, and 6 children answered entirely in Swahili (Table 2). The other 297 children (58%) answered in more than one language; the number of children answering in only one language decreased with age. Across all ages, children answered more expressive vocabulary words in Luo, followed by English and then Swahili. The fraction of expressive responses given in Luo decreased from about 89% among 2‐year‐olds to about 67% among 6‐year‐olds, while the fraction of responses given in English increased from about 5% among 2‐year‐olds to about 29% among 6‐year‐olds (Table 2). The percentage of responses given in Swahili was small (7% among 2‐year‐olds) and decreased slightly with age. The youngest children showed a clear preference for expressing themselves in their mother tongue, as was evident in the patterns of response in our expressive vocabulary test (Figure 3).

#### 3.3.1 Children's vocabulary at baseline and follow‐up

In bivariate analyses, the baseline measure for each language was most strongly associated with the corresponding follow‐up measure (Tables 3, S4 and S5; Models 1–3). When baseline measures of the other languages were included in the analyses (Models 4–6), however, our follow‐up receptive English measure performed differently from other languages in at least two ways: first, English benefited most from the inclusion of baseline measures of the other two languages; second, English was much more strongly associated with the next‐best baseline assessment than were other follow‐up languages (Figure 4). The baseline Luo assessment was also notable for being the next‐strongest correlate of both follow‐up English and follow‐up Swahili.

**Table 3:** The association between baseline receptive vocabulary and follow‐up English receptive vocabulary

Outcome: Follow‐up English receptive vocabulary	e1	e2	e3	e4	e5	e6
	b/se/p	b/se/p	b/se/p	b/se/p	b/se/p	b/se/p
Receptive vocabulary in English (*z‐*score)	0.3554****			0.2811****	0.3035****	0.2593****
	0.0478			0.0477	0.0487	0.0485
	<0.001			<0.001	<0.001	<0.001
Receptive vocabulary in Swahili (*z‐*score)		0.2669****			0.1618****	0.1034**
		0.0472			0.0469	0.048
		<0.001			0.0006	0.0318
Receptive vocabulary in Luo (*z‐*score)			0.3157****	0.2139****		0.1812****
			0.0465	0.0451		0.0463
			<0.001	<0.001		0.0001
Constant	–0.0119	–0.0025	–0.003	–0.0104	–0.0107	–0.0099
	0.0469	0.0483	0.0474	0.0454	0.0458	0.045
	0.7998	0.9585	0.95	0.8187	0.8158	0.8267
*R*‐squared	0.1332	0.0696	0.1009	0.1737	0.1560	0.1821
*N*. of cases	442	442	442	442	442	442

Notes

English, Swahili, and Luo receptive vocabulary are measured using three assessments based on the British Picture Vocabulary Scale (BPVS). Vocabulary scores are age‐adjusted *z‐*scores for children ages 2–6 years. Baseline and follow‐up were conducted approximately 5 weeks apart.

* *p* < 0.1.

** *p* < 0.05.

*** *p* < 0.01.

**** *p* < 0.001.

**Figure 5:**
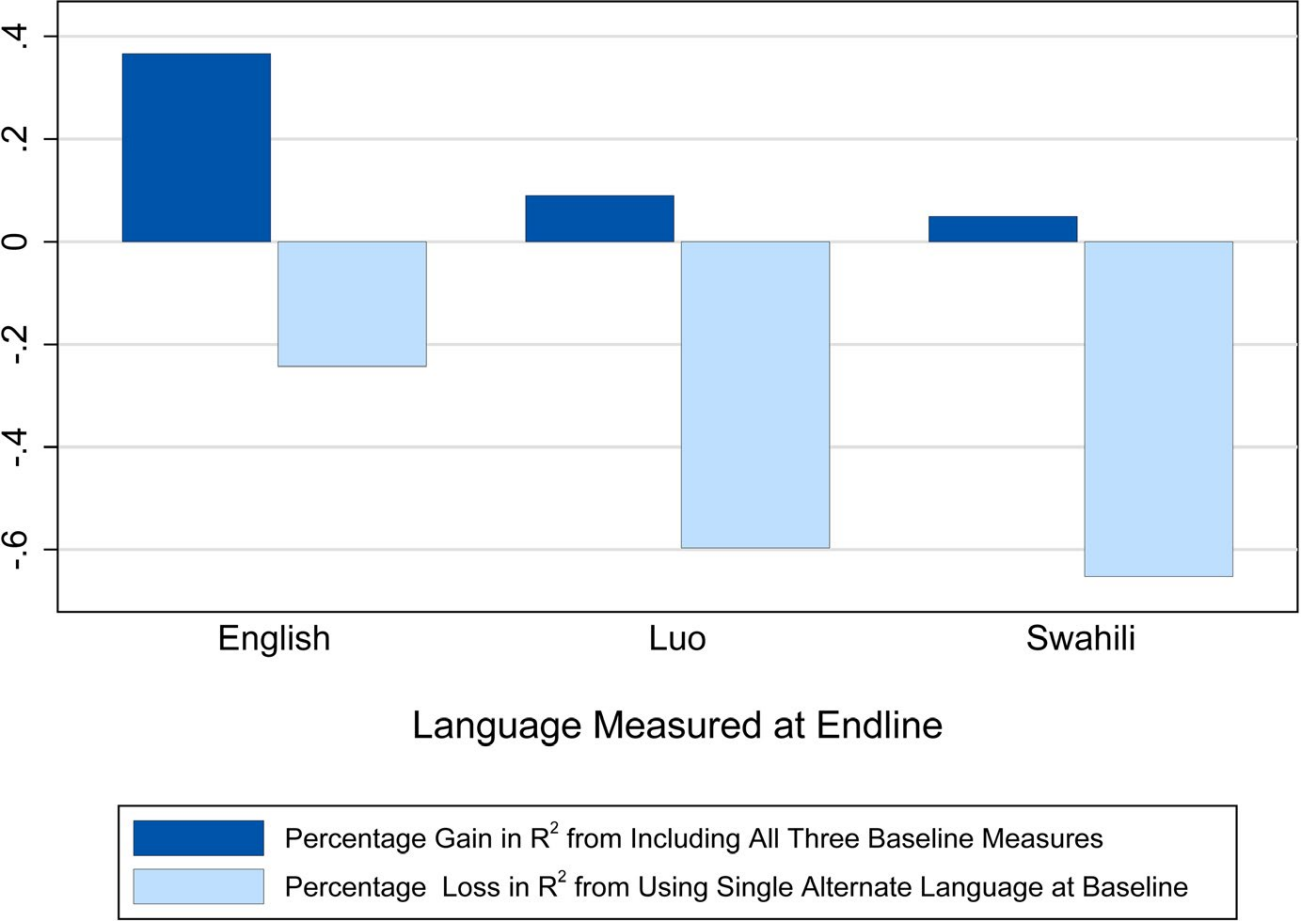
Percentage changes in *R*‐squared relative to test‐retest specification. Notes. Figure depicts changes in *R*‐squared in relation to a regression of follow‐up receptive measures of each language on the baseline measure of the same language. The dark bars shows that the regression of follow‐up English receptive vocabulary on all three languages at baseline yields a 37% increase in *R*‐squared over just using English at baseline, while other languages gain less than 10%. The lighter bars show that the regression of follow‐up English receptive vocabulary on only the next‐most‐strongly associated with baseline language besides itself only reduces the *R*‐squared by 24%, while the next‐best language reduces *R*‐squared by more than 50% for follow‐up measures of languages other than English.

Child baseline Luo receptive vocabulary was significantly associated with follow‐up English receptive vocabulary (*β* = 0.18, *SD* = 0.05, *p* < 0.001), even after accounting for baseline English (*β* = 0.26, *SD* = 0.05, *p* < 0.001) and Swahili (*β* = 0.10, *SD* = 0.05, *p* = 0.032; Model 6) (Table 3). Adding Luo to the English test (moving from Model 1 to Model 4 or 6) increased the *R*‐squared substantially (Figure 5). There was a greater gain in *R*‐squared by testing in both English and Luo (Model 4: *R*‐squared = 0.1737) than English alone, or in English and Swahili (Model 5: *R*‐squared = 0.1560). Testing in all three languages yielded an *R*‐squared of 0.1821 (Model 6). In contrast, baseline receptive vocabulary for all languages was not associated with children's Swahili or Luo vocabulary at follow‐up (Table S4 and S5). Though the other languages were sometimes statistically significant in the full models (Model 6), they did little to increase the overall explanatory power above the bivariate model of only baseline receptive vocabulary scores in the same language (Model 1).

Children's expressive vocabulary at follow‐up was significantly associated with baseline receptive vocabulary measures in all three languages; Luo receptive vocabulary had the strongest association (*β* = 0.26, *SE* = 0.06, *p* < 0.001; Model 8) (Table S6). When baseline expressive vocabulary was included in the full model, however, the receptive vocabulary measures were no longer statistically significant (Model 9).

### 3.4 Associations between caregiver literacy and child vocabulary

In our final analyses, we examined caregiver baseline and child follow‐up measures in English (the primary LOI at older grade levels) and Luo (the mother tongue for 95% of our sample). After adjusting for caregiver education and household wealth, caregiver literacy in Luo was significantly associated with children's receptive vocabulary in English (*β* = 0.11, *SD* = 0.05, *p* = 0.045), while caregiver literacy in English was not (Table 4). Caregiver literacy in either language was not significantly associated with children's receptive vocabulary in Luo or their expressive vocabulary. Moreover, controlling for caregiver literacy (in English and Luo) did not alter the pattern of associations between children's baseline receptive vocabulary (in English, Luo, and Swahili) and follow‐up English receptive vocabulary. Similar patterns were observed among the children of literate and illiterate caregivers, though baseline Swahili receptive vocabulary was more strongly associated with endline English receptive vocabulary among the children of literate caregivers (Table S7).

**Table 4:** The association of baseline caregiver literacy in English and Luo and child follow‐up vocabulary scores

	English receptive vocabulary				Luo receptive vocabulary				Expressive vocabulary			
	cg1	cg2	cg3	cg4	cg5	cg6	cg7	cg8	cg9	cg10	cg11	cg12
	b/se/p	b/se/p	b/se/p	b/se/p	b/se/p	b/se/p	b/se/p	b/se/p	b/se/p	b/se/p	b/se/p	b/se/p
Caregiver literacy												
English	0.070**		–0.057	–0.098	0.0003		–0.040	–0.077	0.049*		0.027	0.021
	0.028		0.073	0.073	0.029		0.088	0.092	0.029		0.081	0.084
	0.013		0.431	0.181	0.993		0.651	0.405	0.088		0.737	0.800
Luo		0.067***	0.109*	0.110**		0.005	0.034	0.028		0.039*	0.019	0.023
		0.022	0.056	0.055		0.023	0.068	0.068		0.022	0.062	0.063
		0.002	0.056	0.045		0.815	0.619	0.680		0.082	0.760	0.711
Constant	–0.190**	–0.234***	–0.224**	–0.237***	–0.003	–0.021	–0.014	–0.039	–0.142	–0.143	–0.148	–0.143
	0.089	0.088	0.090	0.087	0.0936	0.092	0.094	0.093	0.090	0.089	0.090	0.091
	0.034	0.008	0.013	0.007	0.9735	0.822	0.883	0.677	0.117	0.109	0.103	0.118
*R*‐squared	0.015	0.023	0.024	0.045	0	0	0.001	0.016	0.008	0.008	0.008	0.015
*N*. of cases	442	442	442	442	442	442	442	442	442	442	442	442

Notes

Receptive English and Luo vocabulary scores are age‐adjusted *z‐*scores for children ages 2–6 years, measured using separate assessments based on the British Picture Vocabulary Scale (BPVS). Expressive vocabulary *z‐*scores were measured using a tool developed from the PPVT. Caregiver literacy is number of words (out of 5) a caregiver could read from a simple sentence at a second‐grade reading level, adapted from the Early Grade Reading Assessment (EGRA). Baseline and follow‐up were conducted approximately 5 weeks apart. The first two models for each vocabulary assessment (cg1 & cg2, cg5 & cg6, cg9, & cg10) are bivariate regressions. The third model for each vocabulary assessment (cg3, cg7, and cg11) include caregiver literacy in both languages. The fourth model for each vocabulary assessment (cg4, cg8, and cg12) add controls for caregiver education and household wealth.

* *p* < 0.1.

** *p* < 0.05.

*** *p* < 0.01.

**** *p* < 0.001.

## 4 DISCUSSION

In this study, we assessed the language development of 2‐to‐6‐year‐old multilingual children at two time points in a rural, ethnically homogenous region of Kenya. Notably, we found that English receptive vocabulary was less strongly associated with other measures of children's language development than expected, especially among the youngest children. Instead, baseline Luo receptive vocabulary seemed best able to capture general language skill. Specifically, children's baseline Luo receptive vocabulary was significantly associated with English receptive vocabulary at follow‐up, even after taking baseline English receptive vocabulary into account. Luo was also the form of receptive vocabulary most strongly associated with subsequent expressive vocabulary.

Children's follow‐up English receptive scores were significantly associated with their baseline receptive vocabulary scores in all three languages. However, English receptive vocabulary at baseline was not a significant correlate of children's later receptive vocabulary in Swahili or Luo. These findings suggest that, when measuring children's vocabulary at a very young age, an assessment of mother tongue receptive vocabulary provides a strong indication of overall language ability, while LOI receptive vocabulary does not provide a full assessment of vocabulary development. Our findings are consistent with existing work in the study area, in which children demonstrated greater reading fluency in English than in Luo, but significantly lower reading comprehension scores in English than in Luo (Piper, Schroeder, et al., 2016). While children's familiarity with English through their classroom exposure is high, their actual understanding of English is often quite low (Trudell & Piper, 2014). This situation is likely to be common to many African contexts since many children learn to read in a language other than their mother tongue (Lewis et al., 2016).

In the process of vocabulary development, children typically first acquire receptive knowledge of a word (i.e. they recognize and understand the word when it is spoken or read), only later developing the ability to produce the word (expressive vocabulary) either by speaking or writing (Burger & Chong, 2011). By age six, children's receptive vocabulary is usually larger than their expressive vocabulary, although they may also learn to say words before they fully understand them (Burger & Chong, 2011). In our examination of the relationship between children's receptive and expressive vocabulary, we found that the strongest measures of language development at follow‐up were baseline expressive vocabulary (in any language) followed by receptive vocabulary in Luo. However, expressive vocabulary is often not measured in research studies in LMICs—for example, because very young children can be too shy to respond, or may respond correctly in any one of several languages, which makes it more complicated to code responses. Our finding that 59% of children used multiple languages in their expressive responses confirmed our assumption that code‐switching was common.

Caregivers’ literacy in mother tongue at baseline provided an indicator of children's school readiness (as measured by English vocabulary), while caregivers’ English literacy skills at baseline did not. Additionally, caregiver literacy at baseline in either language was not associated with children's vocabulary in Luo at follow‐up. There may be several reasons for these findings. First, young children are often cared for by multiple caregivers, including other children. As a result, their Luo vocabulary may depend less on their primary caregiver's literacy because they hear conversation among other family and caregivers in Luo. Conversely, most families and neighbors do not converse in English, so children would have less regular exposure to the language. Greater caregiver engagement in stimulating activities with their children was associated with higher English, but not Luo receptive vocabulary, suggesting that caregivers may deliberately teach their children English words. Finally, while our measurement of caregiver literacy was designed to be at a second‐grade reading level, it proved more difficult for caregivers in English than in Luo; only three caregivers could read all five words in the English sentence.

A central limitation of this study is that it took place among a rural and ethnically homogenous group of children, so the findings may not generalize to an urban or ethnically mixed setting (Hungi, Njangi, Wekulo, & Ngware, 2017). In mixed ethnicity households or very diverse communities, the associations between mother tongue vocabulary and subsequent LOI vocabulary may not be as strong. However, even within this homogenous group, we had to navigate a multilingual environment to implement language assessments, which presented several inherent challenges. First, items (e.g. “playground”) that perform well in high‐income contexts may be unknown to children in other settings. Additionally, concepts that are represented by a single more difficult word in the original test language may translate to a phrase built from much simpler words: for example, “nest” translates in Luo to *od* (“covering” or “housing”) *winyo* (“bird”) (Capen, 1998), making it an easier word in Luo than the same word in English; thus, the ordering of item difficulty may no longer be appropriate. Finally, even linguistically accurate translations may not retain what some have called “psychological similarity” (van de Vijver & Poortinga, 2005). This is when an item taken from one setting may not have the same psychological meaning in a different context, such as “What do we do before crossing the road?” Therefore, a core strength of our study is the rigorous adaptation, translation, and validation process that we performed for our assessments and our testing of children across a broad age range in multiple languages. This process allowed us to document more fully how children's vocabulary in different languages evolves with age and how receptive vocabulary measures in mother tongue and the LOI were associated with vocabulary development 5–6 weeks later.

In a multilingual context, as is common in LMICs, there is a question of how best to support young children's language and cognitive development. Should pre‐primary educational materials be in the local mother tongue—i.e. children's first language—or in English, the language in which children will eventually be instructed and tested in primary school? Our findings raise the possibility that to best support the language development of children before school age, early childhood interventions—especially those targeting parents—might do well to include instruction and materials in mother tongue, as a child's first language lays the foundation for learning in other languages and for general readiness for school (see also Altan & Hoff, 2018; Hoff & Ribot, 2017).

A recent review of language of instruction policies in Eastern and Southern Africa found that 14 out of 21 countries introduce English as the LOI before fifth grade (Trudell, 2016a). However, it may be particularly challenging in Africa to implement UNESCO's guidelines of at least six years of mother tongue education because of the continent's high degree of linguistic heterogeneity. As a concrete example, Kenya's formal educational policy mandates that early primary instruction be conducted in the mother tongue in rural areas and in Swahili in urban areas––with a transition to English at Grade 4 in either case–– however, this policy is only loosely followed in practice, illustrating the practical challenges inherent in such complex environments (Manyonyi et al., 2016; Trudell, 2016b).

Vocabulary assessment of young children in only one language, particularly if not in their mother tongue, risks inadequately capturing children's development. Foundational work in the study of bilingual education has pointed out the interdependence of language skills across languages for bilingual children, but has focused exclusively on high‐income country examples (e.g. Cummins, 1979). Monolinguals and bilinguals may learn school‐centric words in the LOI equally quickly, but bilingual children may differentially know home‐centric words in their first language rather than the LOI, thereby complicating the interpretation of assessments conducted in a single language (Bialystok et al., 2010). As a specific example of the interplay of languages in Africa, Shin et al. (2015) found that in Malawi, Chichewa literacy in Grade 2 was a predictor of subsequent English skills in Grade 3.

A recent study in Kenya found no additional benefit from mother tongue instruction in primary school on children's language development, but only assessed children's linguistic development in English and Swahili (Piper, Zuilkowski, Kwayumba, & Oyanga, 2018). However, a separate study found that the PRIMR programme (which provides teacher training and instructional supports to improve language and math skills in early primary grades) improved oral reading fluency and reading comprehension in mother tongue (Piper, Zuilkowski, et al., 2016). Our findings suggest that receptive vocabulary in a child's mother tongue may be a particularly important measure of linguistic development, even when the outcome of interest is the language of instruction. Children's vocabulary in their mother tongue may better reflect the level of stimulation and conversation they receive at home, while children's vocabulary in the LOI indicates their exposure to that language. Multilingual testing of parents and children is essential in order to understand the developmental status of multilingual children as well as factors that affect their development in LMICs.

## CONFLICT OF INTEREST

The authors have no conflicts of interest to declare.

## Data Availability

The data that support the findings of this study are available upon reasonable request to oozier@worldbank.org.

## Appendix A

### Receptive Vocabulary Test Translation and Adaptation

To create the receptive vocabulary assessments, we first asked local staff, fluent in all languages, to translate and back‐translate the first 106 items of the British Picture Vocabulary Scale—III (BPVS III; Dunn, Dunn, & Styles, 2009) into Luo and Swahili. The team also evaluated the translated words (for both languages) across various criteria, including (a) does an easily translated, corresponding word exist? (b) is this word commonly known or used? (c) what is the estimated difficulty of this word for young children? (d) is this word common and the same for both rural and urban populations? (e) is the picture representing the stimulus word culturally appropriate and familiar? (f) are the distractor pictures culturally appropriate and familiar? (g) if the stimulus word or picture is not appropriate, which other picture in the plate could be substituted?

This process produced the following results: for nine of the stimulus words, the translation to Luo and Swahili resulted in the same word (for example, ‘money’ is referred to as ‘pesa’ in both languages; ‘airplane’ is ‘ndege’ in both languages); 26 words had no commonly known directly translated equivalent in Luo among adult speakers interviewed; 12 words had no equivalent in Swahili, and 11 had no translation in either Luo or Swahili; 13 Luo and 10 Swahili translations were not single words, but were phrases, or words with a qualifier (e.g. ‘gigantic’ was translated as ‘very big’ in Swahili); five Swahili and four Luo words were appropriated from English, and were identical or nearly identical to the English word; and for 12 stimulus items, the words were suitable for piloting, but the pictures were inappropriate or unfamiliar. Eight plates had words that were more likely to be known in English and were not translated. Luo was the most difficult language to work with, as it had the most limitations in translating the stimulus words, so we created this list first. To increase our capacity to select the best words possible, we (PK, OO) engaged a focus group of six Luo‐speaking mothers and teachers to review 32 words for which we either had no suitable translation, or that were candidate items not part of the original BPVS III.

We piloted 58 items with about 30 Luo‐speaking children 2–6 years of age, and examined pass rates for each word by age group (younger or older than 5 years). Words were then grouped by estimated difficulty level (hard, average or easy), and we sought to have roughly the same number of items at each of the three difficulty levels. As we lacked a sufficient number of Luo words across the three categories, we created some new stimulus words based on results from the focus groups (lantana, bull, roar), providing our own plates of pictures for each. The final Luo test includes 27 items, with approximately nine words in each difficulty level. Of these 27 items, three were new words and pictures, three involved translations including two words (no one‐word translation was known), one used a distractor picture to replace the original stimulus picture (‘boulder’ replaced ‘mountain’), one replaced a plate with more familiar looking pictures, and one slightly changed the stimulus word (from ‘applauding’ to ‘clapping’, as our translators were aware of no distinct word for ‘applauding’.).

We repeated a similar process for creating tests in Swahili (32 items) and English (34 items), each with roughly one‐third of items in the three difficulty categories. The Swahili test included two items altered by changing the stimulus word to a distractor picture, and one item with the stimulus word slightly altered (from ‘sawing’ to ‘cutting’). Nine items were changed for the English test. Five stimulus words were changed to better reflect the English words used for the stimulus picture (e.g. ‘zipper’ was changed to ‘zip’; ‘sedan’ was changed to ‘saloon car’); three distractor pictures replaced original stimulus items; and a new item was introduced, using an existing plate (‘thumb’). With our final set of words we tabulated the rate of correct responses for each item, then sorted the items in descending order by the rate of correct responses.

Based on data collected from the full study population, we used item response theory to assess the content validity of the receptive vocabulary measures. This analysis allowed us to understand the relative difficulty and discrimination of the items, and the equivalency of the receptive vocabulary assessments across the three languages. Of 500 subjects, 71 reached the last (most difficult) Swahili receptive words; 36 reached the most difficult English receptive words; 126 reached the most difficult Luo receptive words; 38 reached the most difficult expressive items The intersection of these four groups was unsurprisingly small: four respondents. The maximum item difficulty was higher for the English test, and the English test had more difficult items (3 items > 4) than the Luo test (no items greater than 3), although the estimated discrimination for all items was lower (0.78 and 1.32 respectively) (Appendix A, Table 5). The Swahili test had more items of higher difficulty than the Luo test (5 items >4), two of which had very high estimated difficulty (floating, 30.60 and blue, 707.01). The Swahili test also had greater overall discrimination than the English test, at 1.22. A two‐parameter model would not converge for the English receptive vocabulary, so only the estimated item difficulty is presented. Likelihood ratio tests for the Swahili and Luo receptive vocabulary tests indicated that for both languages, the two‐parameter model that allowed discrimination to vary by item was a better fit (*p* < .001).

**Table A1:** Receptive Vocabulary Item Difficulty And Discrimination Using IRT (*N* = 505)

English		Swahili			Luo		
BPVS Plate #	Diff	Item (BPVS plate #)	Diff	Discrim	Item	Diff	Discrim
#30	−1.045	kuku (chicken)	−1.202	1.484	guok (dog)	−1.774	2.754
#8	−1.454	mpira (#1)	−0.918	2.445	atudo (#2)	−1.143	1.384
#9	−1.097	kijiko (#5)	−1.425	2.891	dhok (#7)	−0.289	2.219
#6	−0.817	pesa (#12)	−0.918	1.876	rwakruok (#28)	0.121	1.828
#17	1.015	kuruka (#4)	0.484	1.365	ma duong (#42)	−0.283	1.134
talon	1.177	kunywa (#7)	0.415	2.451	pamo (#99)	0.206	1.323
#51	1.629	ndege (#15)	−0.807	2.316	opuk (#16)	−0.187	1.728
#53	0.642	mshipi (#17)	−0.222	1.090	maonge gimoro (#21)	0.047	1.804
waterfall	2.894	kukata (#41)	−0.410	1.075	chiel (#22)	0.670	0.638
#34	1.110	rarua (#61)	0.806	1.166	bwok (#59)	−0.068	1.092
#74	1.380	mkulima (#22)	1.714	0.538	ng'ur (#104)	1.266	0.786
camel	0.388	chemchemi (#38)	0.936	0.899	turubin (#54)	3.122	0.387
#71	2.604	kuogelea (#11)	1.774	0.602	chikruok katolo (#31)	0.641	1.809
#23	0.702	kupima (#58)	1.219	1.098	frimbi (#27)	0.600	1.049
#47	1.453	salamu (#51)	1.044	1.314	jatedo (#65)	0.884	1.284
#48	1.212	ya kugwaruza (#63)	1.109	0.827	pogo (#43)	0.952	1.224
#75	3.436	kuelea (#49)	30.599	0.028	mor (#24)	0.641	1.171
#94	1.174	moto (#14)	−0.112	0.451	ritho (#92)	0.478	2.699
#78	2.335	ngome (#24)	2.479	0.496	osigo (#98)	0.554	2.779
#118	0.940	unganisha (#86)	3.182	0.820	od kich (#84)	0.806	1.200
#19	3.145	mbavu (#76)	8.685	0.177	lwanda (#32)	−1.250	−0.751
#55	3.856	ramani (#56)	−5.889	−0.151	lengo (#50)	2.347	0.779
#79	2.353	daraja (#23)	0.405	0.336	ruath (bull)	2.651	0.441
#69	2.935	samawati (#10)	707.045	0.002	yie (#83)	1.421	1.767
#97	4.408	manyoya (#46)	4.680	0.527	orido (#49)	1.828	1.660
#109	3.055	kiota (#29)	1.154	−0.056	nyabende (lantana)	0.484	0.946
#57	4.408	ndoano (#33)	4.991	0.438	okumbo (#40)	1.356	2.034
#68	1.393	fisi (#82)	3.600	0.328			
#62	3.909	mstatili (#36)	2.049	0.908			
#116	3.909	tawi (#39)	3.277	0.624			
#35	1.937	kisiwa (#73)	3.171	0.657			
#44	4.319						
#70	3.579						
#111	3.300						
Overall Discrim	0.790			1.215			1.321
Model 1<2:	n.a.			*p *< .001			*p* < .001

Note

Estimated using IRT one‐parameter and two‐parameter logistic models. The first parameter is item difficulty, and the second is item discrimination. One‐parameter models estimate an overall discrimination that is held constant for each item. Two‐parameter models allow the item discrimination to vary across items. We used a likelihood ratio test to compare the two models to determine the model with best fit. A two‐parameter model would not converge for the English receptive vocabulary, so only item difficulties were estimated.

## Appendix B

### Expressive vocabulary test creation

To create the expressive vocabulary measure, we began with images from pieces of the BPVS III plates, local storybook illustrations used in a related project, as well as other simple drawings, we presented up to 200 individual pictures to 61 children 2–6 years of age, asking them to name the object or concept the picture showed. We recorded responses in the language (English, Swahili or Luo) children used.

We then reviewed pass rates (in any language) by age (younger or older than 5 years) for each picture, and discarded words with no clear response (e.g., multiple responses for any picture). We again grouped words as hard, average or easy, based on response by age. This resulted in 20 items (scores range from 0–20), and children received credit for responding in any of the three languages. Four items overlapped with those occurring in the receptive tests (two words on the Swahili list, and two on the English list). The rest were a mix of distractors in the BPVS, and stimulus words. A local artist then created all 20 original drawings used in our assessment, displayed below. A child could respond in any of the three languages to score a pass for expressing the word verbally. During instrument development and pretesting, we worked with our field team to identify words that were similar across languages, and understand how they would be differentiated. English and Swahili are not a tonal languages, while Luo is. Thus, for all similar words, the field team agreed on the differences in their pronunciation that would identify a child's response in a given language.

The full expressive vocabulary assessment is provided below, with the intention of making the tool freely available for use for research purposes only. The tool is not meant for, and should not be used for diagnostic purposes. We did not establish any norms. It is also not intended for use as an instructional aid. The images were printed on flashcards, single sided, and with one image per card. Children were asked in their preferred language, “What is this?” for each item. Children were not instructed as to which language to respond in, but responses in any language were accepted. We did not provide further instruction because code‐switching during conversation is very common in this area, and very young children may not be aware which language they are actually speaking for a given word. Thus, a child could respond to each item in the 20‐item test in English, Luo, or Swahili to score a pass for expressing the word verbally. Administration ended with three consecutive fails after the first 10 items.

**Table B1:** Expressive vocabulary assessment

Item	Stimulus Image	Stimulus Word	Accepted Responses		
	Practice Items		English	Swahili	Luo
1		Shoe	Shoe	Kiatu Viatu	Wuoche
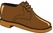
2		Cow	Cow	Ng'ombe	Ruath Dwasi Dhiang
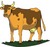
3		Frog	Frog	Chura	Ogwal
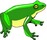
	Test Items				
1		Key	Key	Kifunguo	Ofungu
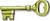
2		Balloon	Baloon	Baluni	Balun
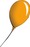
3		Elephant	Elephant	Ndovu	Liech
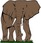
4		Hammer	Hammer	Nyundo	Nyundo
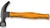
5		Bucket	Bucket	Ndoo	Ndoo
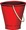
6		Grasshopper	Grasshopper	Panzi	Ongogo
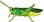
7		Coconut	Coconut tree Palm tree	Mnazi	Nas
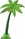
8		Maize	Maize	Mahindi	Oduma Bando
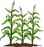
9		Giraffe	Giraffe	Twiga	Twiga
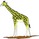
10		Carrot	Carrot	Karoti	Karot
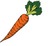
11		Mushroom	Mushroom	Uyoga	Obuolo
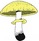
12		Flag	Flag	Bendera	Bandera
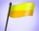
13		Spider	Spider	Buibui	Otieng’ otieng’
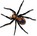
14		Circle	Circle	Mviringo	Sako
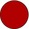
15		Zipper	Zipper	Zippi Zip	Ring’
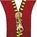
16		Hippo	Hippo	Kiboko	Rao
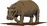
17		Hyena	Hyena	Fisi	Ondiek Otoyo
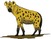
18		Piano	Piano	Kinanda	Kinanda
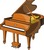
19		Warthog	Warthog	Ngiri	Njiri
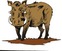
20		Wrench	Wrench Spanner	Spana	Spana
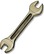

## Appendix C

### MDAT expansion

The adaptation of the MDAT as used in the current study involved multiple steps, drawing from experiences of the study authors (PK, LCHF) using the subscales in other studies and contexts (e.g., author citation redacted). First, we added some items piloted and recommended by the first author of the MDAT (M. Gladstone, personal communication) that had shown good variability in pass rates by age, but were excluded from the final MDAT because they did not meet criteria for inclusion (i.e., they were not passed by 100% of children at the highest age band). Next, we added items that assessed behaviors and knowledge considered important for preparing children for primary school (learning letters, numbers, cardinality, writing letters). We sought to increase the difficulty level of the fine motor/perception subscale by adding Object‐based Pattern Reasoning Assessment (OPRA) items from the Zambia Early Child Development Scale (Zuilkowski, McCoy, Serpell, Matafwali, & Fink, 2016). The concept of the OPRA is similar to two‐dimensional pattern reasoning tests, such as the Ravens Progressive Matrices, but differs in that rather than using pictures, the test uses familiar materials (beans, beads, bottle tops) to make patterns. We added 6 items from the OPRA: two easy items (completing a pattern with one object missing, all of the other objects the same), two medium difficulty items (completing a pattern with two alternating objects), and two hard items (completing a pattern with three alternating objects).

Adaptation of the language subscale included piloting objects used for testing receptive and expressive vocabulary, in order to produce a set of objects that were familiar in the local context. The number of objects presented to the child was increased from 10 to 14, and these included objects easily known by young children (torch, soap) and those that were more challenging (sieve, wick). Finally, as our sample included children 24–35 months of age, we added items from the original MDAT appropriate for testing children at the lower age range. After some adjustments to administration and scoring, the language subscale showed good variability in scores across the age span. We then piloted the fine motor/perception and language subscales with 112 children 36–71 months of age in a nearby, non‐study area in Kenya. We excluded items passed by all children during piloting and dropped a few items with the same difficulty level of other items to reduce the overall length of the subscales. The final adapted test had 21 fine motor/perception items and 26 language items, with start and stop rules to reduce the number of items administered (see Table C1).

**Table C1:** Comparison of original MDAT Language items with Western Kenya MDAT

Item	Fine Motor/Perception	Item	Western Kenya MDAT
1	Follows mother's or guardian's face/object to the midline		Not applicable
2	Follows object or fixes and follows on face or bright object (red pompom) with eyes through 180 degrees.		Not applicable
3	Puts hands together/awareness of hands/puts in front of eyes/mouth		Not applicable
4	Reaches out for a large thing eg. Rattle or red yarn		Not applicable
5	When holding objects, tends to put them in mouth		Not applicable
6	Grasps hold of a large thing e.g. Handle of the rattle or plastic spoon		Not applicable
7	Can pick up a larger object from the ground		Not applicable
8	Can see a small object such as a piece of maize or a bean		Not applicable
9	Transfers objects from one hand to another hand		Not applicable
10	Picks up small things with all four fingers in a RAKING fashion		Not applicable
11	Strikes on object with another in imitation with the examiner		Not applicable
12	Finds object under the cloth		Not applicable
13	Neat pincer grasp, picks up maize or bean with thumb and one finger	START < 36 MONTHS	Neat pincer grasp, picks up maize or bean with thumb and one finger
14	Puts blocks in and out of cup in imitation	14	Puts blocks in and out of cup in imitation
15	Pushes a little car along	15	DELETED
16	Puts blocks into bottle in imitation	16	Puts blocks into bottle in imitation
17	Dumps blocks out of bottle purposefully	17	Dumps blocks out of bottle purposefully
	Screws jar lid on and off	18	Screws jar lid on and off
18	Scribbles on paper (straight scribble)	25	
19	Scribbles on paper (circular scribble)	START 36–59 MONTHS	
20	Tower of 2 blocks	21/24	Puts pegs into board in up to 2 min/30 s
21	Puts pegs into board in up to 2 min	NEW	Makes train with up to 5 blocks
22	Tower of 4 blocks	START 60–71 MONTHS	
23	Tower of 6 blocks	20, 22, 24	Builds tower with up to 6 blocks
24	Puts pegs into board in up to 30 s	30	Can make a bridge with 3 blocks**FOR ALL: SKIP TO HEAVIEST BOX IF FAIL**
25	Unscrews and screws back on the cap of the Chiponde bottle	NEW	Can make a bridge with 6 blocks
26	Can put 6 hair beads on to a shoe lace (thread them on)	NEW	Can make stairs with 6 blocks
27	Copies a vertical line ( as drawn by the examiner) with charcoal/chalk within 30 degrees	29	Picks heaviest box
28	Picks longest stick 3 of 3	18/19	Scribbles in any way
29	Picks heaviest box 3 of 3—is the child able to tell you which box is the heaviest?		**IF≥36 MONTHS AND FAILS: SKIP TO FOLDS **
30	Can make a bridge with bricks:		**END IF FAILS SMALL BRIDGE, HEAVIEST BOX AND SCRIBBLES**
31	Makes a doll or complicated car out of clay	27	Copies a vertical line within about 30 degrees
32	Copies a circle (needs to be complete) with chalk or in the sand with a stick	34	Copies a circle
33	Copies a cross with chalk	33	Copies a cross
34	Can draw a square		**END IF FAILS LINE, CIRCLE, CROSS**
35		NEW	Can color within lines
36		34	Copies a square
37		NEW	Can copy letters **E C A M J H**
38		NEW	Can fold paper into quarters
39			**END IF FAILS 3 OF ABOVE (COLORING, SQUARE, WRITING 4 LETTERS AND FOLDING)**
40		NEW	Can copy a pattern of 4 bottle tops **END IF FAILS 4 BOTTLE TOPS**
41		NEW	Can copy a pattern of 6 bottle tops **END IF FAILS 6 BOTTLE TOPS**
42		NEW	Beans and beads
43		43	

**Table C2:** Comparison of original MDAT Language items with Western Kenya MDAT

Item	Language/Hearing	Item	Western Kenya MDAT
	Startles or jumps/responds to sounds		
**1**	Happy vocalising or making sounds—not crying		
2	Laughs/chuckles		
3	Turns to voice—if you are out of sight, does she/he look in the direction of your voice or sound		
4	Uses single syllables or sounds, for example Ma, Pa, Da, Ba		
5	Responds to his or her name, turns and looks at you		
6	Uses 2/4 syllable babble such as dada, mama, mimi, tata, but not specifically at anything or any person		
7	Understands when being cautioned about danger, for example when saying “no” to child, they stop even briefly		
8	Indicates by gesture to say “No”	START < 36 MONTHS STOP RULE: IF 3 OF LAST FIVE ADMINISTERED ARE FAILED, STOP	Child shakes head or uses other gesture when means “no” (ask parent/caregiver if not observed)
9	Follows simple commands (1 stage)	10	Child jabbers: makes sentence‐like utterances, even if cannot use all real words (ask parent/caregiver if not observed). If child already speaks in sentences at least some of the time, score YES.
10	Unclear talk/jabber in sentences	11, 13	Child uses **6 or more** words (ask parent/caregiver if not observed)
11	Says 2 words, but words other than mama/dada	12	Child uses 2 or more words together to form some type of phrase with subject/object verb (“Mama go”) (ask parent/caregiver if not observed)
12	Says 2 words together	16	Child speaks in sentences at least some of the time (ask parent/caregiver if not observed)
13	Says 6 words (words other than mama/dada)	9	Child follows one stage command. Can do up to 2 times (differential scoring based on which trial passed: 2 = passed first time, 1 = passed second time, 0 = failed all times)
14	Follows 2 stage commands		START 36–59 MONTHS STOP RULE: IF 3 OF LAST FIVE ADMINISTERED ARE FAILED, STOP
15	Identifies objects in the basket—at least 5	19	Can tell you first name or nickname—how child is known (can be observed incidentally or if not observed, ask child to tell you name) This will be asked at beginning of FM and if fails, asked again here.
16	Speaks clearly in sentences	17	Can point to 5 or more body parts (YES/NO) Record all pointed to. Head, toes, tongue, hard. Move nose eyes ears or mouth (1) to language test—eyes hair and hand to language test with cup pencil and comb.
17	Points to body parts > 1	14	Follows 2 stage command. Can do up to 2 times (differential scoring based on which trial passed: 2 = passed first time, 1 = passed second time, 0 = failed all times)
18	Names 5 objects in the basket	18	Can **name** 5 or more objects you point to if under 36 months. Otherwise, must name 10 or more objects you point to to pass. Record all named
19	Knows his or her first name	15	Can **identify** (point to) 5 or more objects you name if under 36 months. Otherwise, must identify 10 or more objects you point to to pass. Record all identified
20	Knows actions of objects		START 60–71 MONTHS STOP RULE: IF 3 OF LAST FIVE ADMINISTERED ARE FAILED, STOP
21	Identifies objects—10	20	Can **point** to 3 or more objects linked to action (“Which one is for sweeping?”) Record all identified
22	Names (can say it) 10 objects	25	Can **tell** you what 3 or more objects are used for (“For washing”) Record all verbal descriptions
23	Is able to categorise things	23	Can list 4 + foods Record all foods named OR
24	Is able to follow a three stage command	23	Can list 4 + animals Record all animals named
25	Is able to tell you the use of objects	24	Can perform a 3‐stage command Can do 1 time. Child must do correctly to PASS
26	Can do remember back 2 syllables	27	Can answer 2 or more questions about what to do in certain situations (hungry, tired, cold)
27	Knows 2 of 3 questions relating to the understanding certain concepts	28	Correctly answers questions about BOTH adjectives (faster, bigger)
28	Understands the adjectives such as “faster”	30	Knows 3 OR MORE prepositions and follows tasks related to this (under, on, between, etc.)
29	Can do remember back 4 syllables	31	Understands and passes 2 OR MORE concepts of opposites
30	Can understands prepositions	32/33	Can count to 5 correctly
31	Understands the concept of opposites	NEW	Can count to 10 correctly
32	Knows quantities—up to 3	NEW	Can create sets of objects (1, 3 or 5) PASS IF CHILD CAN DO 2 OF THESE
33	Knows quantities—up to 5	NEW	Can name 2 colors
34		NEW	Can recognize and name 3 or more written letters in first name
35		NEW	Knows age
36		NEW	Knows where they currently live
