# Supplementary material for: Multilingual assessment of early child development: Analyses from repeated observations of children in Kenya
Source: Dev Sci. 2019 Jul 28;22(5):e12875. doi: 10.1111/desc.12875 (PMC6771616; doi:10.1111/desc.12875)
Supplement: Supplementary file 1 [file DESC-22-na-s001.docx]

Supplemental Table 1. Internal consistency and interrater reliability of child development measures

|  | Obs. | No. items in scale | Cronbach's alpha | Interrater reliability N = 48 | | |
| --- | --- | --- | --- | --- | --- | --- |
|  |  |  |  | Percent agreement | Cohen's kappa | Krippendorff's alpha |
| Receptive vocabulary | |  |  |  |  |  |
| English | 505 | 34 | 0.569 | 0.958333 | 0.954023 | 0.998914 |
| Swahili | 505 | 31 | 0.757 | 0.895833 | 0.888424 | 0.996739 |
| Luo | 505 | 27 | 0.779 | 1 | 1 | 1 |
| Expressive vocabulary | 505 | 20 | 0.660 | 0.958333 | 0.949712 | 0.99934 |
| Adapted MDAT |  |  |  |  |  |  |
| Fine motor | 495 | 32 | 0.935 | 0.9375 | 0.934005 | 0.999635 |
| Language | 495 | 28 | 0.901 | 0.875 | 0.864407 | 0.996617 |
| Notes. English, Swahili, and Luo receptive vocabulary were measured using separate assessments based on the British Picture Vocabulary Scale (BPVS). Expressive vocabulary was measured using a tool developed from the PPVT. The adapted MDAT is the Kenya adaptation of the Malawi Developmental Assessment Tool (MDAT). | | | | | | |

Supplemental Table 2. Correlation matrix of baseline age-standardized child development measures

|  | Receptive vocabulary | | |  | Adapted MDAT | |
| --- | --- | --- | --- | --- | --- | --- |
|  | English | Swahili | Luo | Expressive vocabulary | Fine motor | Language |
| Receptive vocabulary |  |  |  |  |  |  |
| English | 1 |  |  |  |  |  |
| Swahili | 0.358958 | 1 |  |  |  |  |
| Luo | 0.364414 | 0.400669 | 1 |  |  |  |
| Expressive vocabulary | 0.43381 | 0.434502 | 0.490148 | 1 |  |  |
| adapted MDAT Fine motor | 0.323954 | 0.430528 | 0.490547 | 0.447659 | 1 |  |
| adapted MDAT language | 0.421838 | 0.319749 | 0.476982 | 0.465957 | 0.555477 | 1 |
| Notes. English, Swahili, and Luo receptive vocabulary are *z-*scores, measured using separate assessments based on the British Picture Vocabulary Scale (BPVS). Expressive vocabulary *z-*scores were measured using a tool developed from the PPVT. The adapted MDAT are the *z-*scores from the Kenya adaptation of the Malawi Developmental Assessment Tool (MDAT). All *z-*scores were age adjusted. All correlations are significant at *p* < 0.001. | | | | | | |

Supplemental Table 3. Association between contextual factors and child development scores at baseline using bivariate OLS regression

|  | Receptive vocabulary | | | | | | Expressive vocabulary | | Adapted MDAT | | | |
| --- | --- | --- | --- | --- | --- | --- | --- | --- | --- | --- | --- | --- |
|  | English | | Swahili | | Luo | |  |  | Fine motor | | Language | |
|  | b | se | b | se | b | se | b | se | b | se | b | se |
| *Child characteristics:* |  |  |  |  |  |  |  |  |  |  |  |  |
| Height-for-age *z-*score | 0.25*** | 0.03 | 0.19*** | 0.03 | 0.26*** | 0.04 | 0.3*** | 0.04 | 0.34*** | 0.03 | 0.33*** | 0.03 |
| *Primary caregiver characteristics:* |  |  |  |  |  |  |  |  |  |  |  |  |
| Caregiver completed primary school | 0.19* | 0.09 | 0.22** | 0.1 | 0.15 | 0.09 | 0.29*** | 0.1 | 0.23** | 0.1 | 0.15 | 0.1 |
| Caregiver is literate | 0.13 | 0.1 | 0.11 | 0.1 | 0.03 | 0.09 | 0.23** | 0.1 | 0.18* | 0.1 | 0.19* | 0.1 |
| Caregiver working memory (out of 20) | 0.08*** | 0.03 | 0.04 | 0.02 | 0.03 | 0.02 | 0.06** | 0.03 | 0.05* | 0.03 | 0.07** | 0.03 |
| Caregiver depressive symptoms (out of 26) | . | 0.01 | . | 0.01 | –0.01 | 0.008 | 0.01 | 0.01 | –0.01 | 0.01 | 0.01 | 0.01 |
| *Household characteristics:* |  |  |  |  |  |  |  |  |  |  |  |  |
| Household size | –0.11 | 0.05 | –0.08 | 0.05 | –0.15*** | 0.05 | –0.06 | 0.05 | –0.07 | 0.06 | –0.03 | 0.05 |
| Household wealth index | 0.03 | 0.02 | 0.04** | 0.02 | –0.01 | 0.02 | 0.04** | 0.02 | 0.05** | 0.02 | 0.02 | 0.02 |
| Family care indicators score (out of 17) | 0.03* | 0.01 | 0.02 | 0.01 | 0.01 | 0.01 | 0.03* | 0.02 | 0.05*** | 0.01 | 0.04** | 0.01 |
| Notes. Receptive English and Luo vocabulary scores are *z-*scores, measured using separate assessments based on the British Picture Vocabulary Scale (BPVS). Expressive vocabulary *z-*scores were measured using a tool developed from the PPVT. The adapted MDAT are the *z-*scores from the Kenya adaptation of the Malawi Developmental Assessment Tool (MDAT). All *z-*scores were age adjusted. Baseline and follow-up were conducted approximately 5 weeks apart. **p* < 0.1, ***p* < 0.05, ****p* < 0.01. | | | | | | | | | | | | |

Supplemental Table 4. The association between baseline receptive vocabulary and follow-up Swahili receptive vocabulary

|  | s1 | s2 | s3 | s4 | s5 | s6 |
| --- | --- | --- | --- | --- | --- | --- |
|  | b/se/p | b/se/p | b/se/p | b/se/p | b/se/p | b/se/p |
| Receptive vocabulary in English (*z-*score) | 0.2214**** |  |  |  | 0.0675 | 0.0431 |
|  | 0.0452 |  |  |  | 0.0413 | 0.0448 |
|  | <0.001 |  |  |  | 0.1036 | 0.3372 |
| Receptive vocabulary in Swahili (*z-*score) |  | 0.5031**** |  | 0.4578**** | 0.4798**** | 0.4476**** |
|  |  | 0.0419 |  | 0.0461 | 0.0452 | 0.0473 |
|  |  | <0.001 |  | <0.001 | <0.001 | <0.001 |
| Receptive vocabulary in Luo (*z-*score) |  |  | 0.2912**** | 0.1114** |  | 0.0998* |
|  |  |  | 0.0481 | 0.0473 |  | 0.0509 |
|  |  |  | <0.001 | 0.0191 |  | 0.0508 |
| Constant | –0.0064 | –0.0011 | –0.0011 | –0.0013 | –0.0029 | –0.0024 |
|  | 0.0475 | 0.0406 | 0.0464 | 0.0405 | 0.0406 | 0.0405 |
|  | 0.8923 | 0.9793 | 0.9805 | 0.9747 | 0.9436 | 0.9524 |
| *R*-squared | 0.0508 | 0.2429 | 0.0843 | 0.2532 | 0.2471 | 0.2548 |
| N. of cases | 442 | 442 | 442 | 442 | 442 | 442 |
| Notes. Receptive English, Swahili, and Luo vocabulary scores are age-adjusted *z-*scores for children ages 2 to 6 years, measured using separate assessments based on the British Picture Vocabulary Scale (BPVS). Baseline and follow-up were conducted approximately 5 weeks apart. **p* < 0.1, ***p* < 0.05, ****p* < 0.01, *****p* < 0.001. | | | | | | |

Supplemental Table 5. The association between baseline receptive vocabulary and follow-up Luo receptive vocabulary

|  | l1 | l2 | l3 | l4 | l5 | l6 |
| --- | --- | --- | --- | --- | --- | --- |
|  | b/se/p | b/se/p | b/se/p | b/se/p | b/se/p | b/se/p |
| Receptive vocabulary in English (*z-*score) | 0.2780**** |  |  | 0.1125** |  | 0.0857* |
|  | 0.052 |  |  | 0.0445 |  | 0.0461 |
|  | <0.001 |  |  | 0.0121 |  | 0.0641 |
| Receptive vocabulary in Swahili (*z-*score) |  | 0.3340**** |  |  | 0.1472**** | 0.1269*** |
|  |  | 0.0481 |  |  | 0.0437 | 0.0455 |
|  |  | <0.001 |  |  | 0.0008 | 0.0056 |
| Receptive vocabulary in Luo (*z-*score) |  |  | 0.5168**** | 0.4761**** | 0.4590**** | 0.4359**** |
|  |  |  | 0.0443 | 0.0474 | 0.0474 | 0.0492 |
|  |  |  | <0.001 | <0.001 | <0.001 | <0.001 |
| Constant | –0.01 | –0.0028 | –0.0037 | –0.0067 | –0.0038 | –0.0061 |
|  | 0.049 | 0.0477 | 0.0423 | 0.0424 | 0.0419 | 0.0421 |
|  | 0.8383 | 0.9524 | 0.9295 | 0.8742 | 0.9279 | 0.8857 |
| *R*-squared | 0.0797 | 0.1066 | 0.2645 | 0.2759 | 0.2819 | 0.2882 |
| N. of cases | 442 | 442 | 442 | 442 | 442 | 442 |
| Notes. Receptive English, Swahili, and Luo vocabulary scores are age-adjusted *z-*scores for children ages 2 to 6 years, measured using separate assessments based on the British Picture Vocabulary Scale (BPVS). Baseline and follow-up were conducted approximately 5 weeks apart. **p* < 0.1, ***p* < 0.05, ****p* < 0.01, *****p* < 0.001. | | | | | | |

Supplemental Table 6. The association between receptive vocabulary at baseline and expressive vocabulary at follow-up

|  | x1 | x2 | x3 | x4 | x5 | x6 | x7 | x8 | x9 |
| --- | --- | --- | --- | --- | --- | --- | --- | --- | --- |
|  | b/se/p | b/se/p | b/se/p | b/se/p | b/se/p | b/se/p | b/se/p | b/se/p | b/se/p |
| Receptive vocabulary in English (*z-*score) | 0.3236*** |  |  |  |  | 0.2361*** | 0.2127*** | 0.1728*** | 0.0347 |
|  | 0.0541 |  |  |  |  | 0.0517 | 0.0468 | 0.0473 | 0.0375 |
|  | <0.01 |  |  |  |  | <0.01 | <0.01 | 0.0003 | 0.3547 |
| Receptive vocabulary in Swahili (*z-*score) |  | 0.3545*** |  |  | 0.2300*** | 0.2727*** |  | 0.1891*** | 0.0108 |
|  |  | 0.0528 |  |  | 0.0505 | 0.0518 |  | 0.052 | 0.0371 |
|  |  | <0.01 |  |  | <0.001 | <0.001 |  | 0.0003 | 0.7721 |
| Receptive vocabulary in Luo (*z-*score) |  |  | 0.3961*** |  | 0.3058*** |  | 0.3191*** | 0.2592*** | 0.0217 |
|  |  |  | 0.0571 |  | 0.0579 |  | 0.0542 | 0.0552 | 0.0386 |
|  |  |  | <0.01 |  | <0.001 |  | <0.001 | <0.001 | 0.5748 |
| Expressive vocabulary (*z-*score) |  |  |  | 0.7385*** |  |  |  |  | 0.7084*** |
|  |  |  |  | 0.0409 |  |  |  |  | 0.0564 |
|  |  |  |  | <0.001 |  |  |  |  | <0.001 |
| Constant | –0.0176 | –0.0092 | –0.0098 | –0.0019 | –0.0098 | –0.0156 | –0.0154 | –0.0144 | –0.0032 |
|  | 0.0472 | 0.0457 | 0.0461 | 0.0314 | 0.0439 | 0.0441 | 0.0447 | 0.0431 | 0.0316 |
|  | 0.7094 | 0.8405 | 0.8327 | 0.9529 | 0.823 | 0.7242 | 0.731 | 0.7387 | 0.9205 |
| *R*-squared | 0.1115 | 0.1239 | 0.1603 | 0.5538 | 0.2042 | 0.1767 | 0.2024 | 0.2306 | 0.5558 |
| N. of cases | 442 | 442 | 442 | 442 | 442 | 442 | 442 | 442 | 442 |
| Notes. Receptive English, Swahili, and Luo vocabulary scores are age-adjusted *z-*scores for children ages 2 to 6 years, measured using separate assessments based on the British Picture Vocabulary Scale (BPVS). Expressive vocabulary *z-*scores were measured using a tool developed from the PPVT. Baseline and follow-up were conducted approximately 5 weeks apart.  **p* < 0.1, ***p* < 0.05, ****p* < 0.01, *****p* < 0.001. | | | | | | | | | |

Supplemental Table 7. Relationships between baseline receptive vocabulary and follow-up English receptive vocabulary among children of literate and illiterate caregivers

|  | e1 | e2 | e3 | | e4 | e5 | | e6 |
| --- | --- | --- | --- | --- | --- | --- | --- | --- |
|  | b/se/p | b/se/p | b/se/p | | b/se/p | b/se/p | | b/se/p |
|  |  | | | Luo literacy | | | English literacy | |
| Sample (above/below median literacy): | Both | Both | Below | | above | Below | | above |
| Receptive vocabulary in Luo (*z-*score) | 0.1812*** | 0.1788*** | 0.1412* | | 0.1925*** | 0.1511** | | 0.1932*** |
|  | 0.0463 | 0.046 | 0.083 | | 0.0547 | 0.0732 | | 0.0592 |
|  | 0.0001 | 0.0001 | 0.0915 | | 0.0005 | 0.0411 | | 0.0013 |
| Receptive vocabulary in English (*z-*score) | 0.2593*** | 0.2515*** | 0.3113*** | | 0.2288*** | 0.3391*** | | 0.2128*** |
|  | 0.0485 | 0.0488 | 0.0914 | | 0.0562 | 0.0802 | | 0.0594 |
|  | <0.001 | <0.001 | 0.0009 | | 0.0001 | <0.001 | | 0.0004 |
| Receptive vocabulary in Swahili (*z-*score) | 0.1034** | 0.0998** | 0.0564 | | 0.1316** | 0.052 | | 0.1336** |
|  | 0.048 | 0.0481 | 0.0764 | | 0.0613 | 0.0698 | | 0.066 |
|  | 0.0318 | 0.0386 | 0.4623 | | 0.0331 | 0.4576 | | 0.0446 |
| Caregiver literacy in Luo |  | 0.0639 |  | |  |  | |  |
|  |  | 0.0429 |  | |  |  | |  |
|  |  | 0.1376 |  | |  |  | |  |
| Caregiver literacy in English |  | –0.018 |  | |  |  | |  |
|  |  | 0.0546 |  | |  |  | |  |
|  |  | 0.7416 |  | |  |  | |  |
| Constant | –0.0099 | –0.1825** | –0.1697** | | 0.0801 | –0.0986 | | 0.0549 |
|  | 0.045 | 0.0776 | 0.0729 | | 0.0568 | 0.0681 | | 0.0594 |
|  | 0.8267 | 0.0193 | 0.0218 | | 0.1605 | 0.1501 | | 0.3569 |
| *R-*squared | 0.1821 | 0.1951 | 0.1833 | | 0.1796 | 0.2149 | | 0.1656 |
| N. of cases | 442 | 442 | 158 | | 284 | 187 | | 255 |
| Notes. Receptive English, Swahili, and Luo vocabulary scores are age-adjusted *z-*scores measured using separate assessments based on the British Picture Vocabulary Scale (BPVS). Baseline and follow-up were conducted approximately 5 weeks apart. **p* < 0.1, ***p* < 0.05, ****p* < 0.01. | | | | | | | | |
